# Cumulative Risk of Immigration Prison Conditions on Health Outcomes Among Detained Immigrants in California

**DOI:** 10.1007/s40615-021-01187-1

**Published:** 2021-11-29

**Authors:** Altaf Saadi, Caitlin Patler, Maria-Elena De Trinidad Young

**Affiliations:** 1 Department of Neurology, Massachusetts General Hospital, Harvard Medical School, 100 Cambridge St, Suite 2000, Boston, MA 02114 USA; 2 Department of Sociology, UC Davis, 1283 Social Sciences & Humanities, One Shields Ave, Davis, CA 95616 USA; 3 Department of Public Health, School of Social Sciences, Humanities, and Arts, University of California, Merced, 5200 N. Lake Rd, Merced, CA 95343 USA

**Keywords:** Immigration detention, Immigrant health, Incarceration, Immigration policy

## Abstract

**Objectives:**

The USA maintains the world’s largest immigration detention system. This study examines the mechanisms by which detention serves as a catalyst for worsening health.

**Methods:**

Using data from detained immigrants in California (*n* = 493) from 2013 to 2014, we assessed the prevalence of exposure to conditions of confinement hypothesized to negatively influence health; the extent to which conditions of confinement are associated with psychological stress, diagnosed mental health conditions, and/or declines in general health; and the cumulative impact of confinement conditions on these outcomes.

**Results:**

We found that each condition increased the likelihood of one or more negative health conditions, but there was also a cumulative effect: for each additional confinement condition, the odds of worsening general health rose by 39% and reporting good health decreased by 24%.

**Conclusions:**

Confinement conditions are associated with poor physical and mental health outcomes among immigrants detained in immigration prisons. Policies that seek to improve specific conditions in detention centers may remove some risks of harm, but alternatives to detention are likely to be most effective.

## Introduction

The USA is the world leader in immigration detention, with the highest number of imprisoned immigrants worldwide [1]. Between 2008 and 2018, ICE apprehended over two million noncitizens in the interior of the country [2]. About one in four Latinos in the USA report knowing someone who had been deported or detained by immigration enforcement within the last year [3]. Yet, we know little about the health status and outcomes of immigrants in the United States (U.S.). who experience detention (also referred to as immigration prison), due in large part to a dearth of data and lack of data transparency from U.S. Immigration and Customs Enforcement (ICE) and other immigration enforcement agencies about the health of individuals in their custody. Recent immigrant health research shows that anti-immigrant policies and immigration enforcement (e.g., workplace raids, police collaboration with ICE) are associated with poor health outcomes [4]. Immigration imprisonment resides along the continuum of migration-related social and structural determinants of health, including pre-migratory trauma and post-migratory trauma such as racism that portend long-term adverse consequences for health [5]. Understanding the health impacts of immigration prisons is a critical component of advancing health equity among immigrants.

Research among U.S. populations incarcerated under criminal law demonstrates that individuals’ health status declines as a result of prisons’ physical conditions and practices [6]. Prison conditions and practices can confer both direct and indirect harms to health: directly through increased exposures to infectious diseases or inadequate nutrition, or indirectly via interruptions in health care or substandard care contributing to new illness or, over the long term, exacerbating existing illness [7]. Prison conditions also expose individuals to punitive practices, such as 24-hour lighting and resultant sleep deprivation, solitary confinement, physical and psychological abuse, and severing of social connections, associated with worsening stress, mental health, and physical health outcomes [6, 7]. We refer to these prison conditions and practices that can negatively impact health as “conditions of confinement,” as they are inherent to the design and operation of prisons both in the criminal and immigration contexts.

Immigration prisons have similar conditions as other prisons, exposing people to similar physical and emotional harms [8]. This is despite falling within the legal purview of civil rather than criminal law and legally defined as a nonpunitive administrative holding process. Human rights and other legal groups have documented how immigration prison regulations and oversight (or lack therefore) have resulted in systemic medical neglect; physical, sexual, and verbal abuse; and preventable deaths [9, 10]. In the only existing study of detained immigrants’ health, over two in five individuals detained in California reported a diagnosed chronic health condition, and one in five of those individuals reported an interruption in their medical care while detained [11].

Our study is aimed at assessing the extent to which immigrants’ experiences of both individual and cumulative immigration prison conditions may influence the health of detained individuals. The conditions of confinement we examine include sleep deprivation, social isolation from family via difficulty accessing family visitation, witnessing or experiencing abuse or harassment, and barriers to needed physical and mental health care. We draw on a sample of U.S.-based detained immigrants in California to assess (1) the prevalence of exposure to these conditions of confinement; (2) the extent to which conditions of confinement are associated with psychological stress, diagnosed mental health conditions, and/or declines in general health; and (3) the cumulative impact of the conditions on these outcomes.

## Methods

### Study Design and Sample

We analyzed cross-sectional survey data from the Rodriguez Survey (RS), the only existing survey of detained people in the U.S. that captures information about health and conditions of confinement [11]. Participants were drawn from a census of individuals detained in one of four immigration prisons in the Central Federal Court District of California who were members of the *Rodriguez v. Robbins* class action litigation establishing the right to bond hearings for most individuals held in ICE custody for 180 days or longer (715 F.3d 1127 (9th Cir. 2013)).

The RS was conducted in 2013–2014 using a 92-question in-person survey, administered in English or Spanish, to 565 detained *Rodriguez* class members who were ≥ 18 years old (participation rate = 92%). The RS survey instrument was adapted from a survey of individuals incarcerated under criminal law [12, 13]. The RS gathers information about demographic background; work, criminal, immigration, and legal history; family and household; physical and mental health; and experiences within jails and immigration prisons. Respondents did not receive compensation. The sample is similar to the population of individuals in prolonged immigration prison in California at the time of the study (see Appendix for details). The RS contained small amounts of missing data, ranging from 0 to 4.6% across our outcome and control variables. After casewise deletion, the effective sample for this study was 493 individuals (87.3% of the original sample).

### Measures of Conditions of Confinement

We created dichotomous variables (yes = 1, no = 0) identifying if respondents had experienced the following: (1) sleep deprivation; (2) difficulty accessing family visitation; (3) harassment (including experiencing formal discipline and physical or verbal abuse by a guard or staff member); (4) witnessed harassment (including physical or verbal abuse by a guard or staff member); (5) difficulty accessing medical services; and (6) difficulty accessing counseling or psychological services. We then created a sum index of the six confinement conditions.

For confinement conditions 1, 2, 5, and 6, respondents were asked using a 4-point Likert scale to rate the difficulty sleeping, seeing family members, accessing medical services, and accessing counseling or psychological services. We grouped respondents as experiencing difficulty (= 1) if they answered “difficult” or “very difficult” versus “easy” and “very easy” = 0. Confinement condition 3 was defined using the question, “Have you ever been punched or assaulted by a guard or staff member. This includes, for example, being hit, shoved, or spat upon.” We defined verbal abuse from the following question: “Have you ever been verbally insulted, humiliated, or threatened by a guard or staff member?” (yes = 1, no = 0). Witnessing physical or verbal abuse (condition 4) includes respondents’ answers to whether they have seen/witnessed another detained individual experiencing physical or verbal abuse as defined above.

### Health Outcome Measures

First, we created an index of stress symptoms, adapted from the American Psychological Association annual “Stress in America” survey [14]. Respondents were asked if they have experienced any of the following in the past month (yes = 1, no = 0): headaches; frequent upset stomach or indigestion; frequently feeling as though you could cry; muscular tension; feeling nervous or sad; irritability or anger; inability to sleep or sleeping too much; lack of interest, motivation, or energy; fatigue; teeth grinding; and change in appetite. We summed responses into an index of stress symptoms (range, 0–11). Second, we created a variable categorizing individuals as having ever received a diagnosis of a mental health condition from a healthcare professional (yes = 1, no = 0). Third, we created a dichotomous measure of good overall general health. Respondents were asked, “compared to others your age, how would you describe your current overall physical health? Excellent good, fair, or poor?” (excellent/good = 1, fair/poor = 0). Fourth, we created a dichotomous variable assessing respondents’ perceptions of whether their health worsened during detention (“How do you think your current physical health compares to your health before immigration detention?” Health was better before detention = 1; health is better now, or health is about the same = 0).

### Covariates

We adjusted for background characteristics that may be associated with detained immigrants’ health, including self-reported sex (male vs. female), age, educational attainment (high school degree/GED or more vs. less than high school), ethnicity (Hispanic/Latina/o vs. any other), English language proficiency (speaks English very well/pretty well vs. just a little/not at all), immigration status (undocumented vs. any other status), pre-detention health insurance (yes/no), detention facility, months detained by ICE at time of survey, and longest previous incarceration (in months, natural-logged to account for overdispersion or increased variability in the data), if any.

### Statistical Analysis

We conducted univariate analyses of all variables. We then conducted multivariable regression to assess the association between the conditions of confinement measures and the outcomes, adjusting for covariates. We used logistic regression for models assessing overall health, worse health in detention, and diagnosis of mental health conditions (Models 1–3, yielding an odds ratio). We used Poisson regression to assess the index of stress symptoms (Model 4, yielding an incidence rate ratio). Our substantive results did not change when we allowed the sample size to vary versus limiting to the sample without missing data (*n* = 493) (results not shown). We conducted all analyses in Stata 16.

## Results

Of 493 respondents, 91.7% were male, with an average age of 37.1 years old (SD 9.2, range 18.6–68.9) (Table 1); 43.4% had a high school diploma, GED, or higher; and 52.7% spoke English well. Almost 3 in 4 respondents (71.8%) were undocumented, and the remaining 28.2% had a visa or permanent residency at the time of their apprehension by ICE. Less than half (41%) had health insurance prior to detention. Respondents were held across four facilities, with 43% housed in a private, for-profit facility. On average, respondents had spent 8.7 months detained at time of interview (SD 4.7, range 3.3–55.5). Respondents had also spent an average of 22 months incarcerated in the criminal justice system prior to detention (SD 47.6, range 0–600 months).

**Table 1:** Sample characteristics (*n*** = **493)

Variable	Mean (%)	SD	Min	Max
*Health outcomes*				
Good overall health	69.37			
Diagnosed with depression or other mental health condition	16.43			
Worse health in detention	55.98			
Count of stress symptoms	5.84	3.20	0.00	11.00
*Conditions of confinement*				
Experienced sleep deprivation	74.85			
Difficulty seeing family	37.93			
Experienced verbal or physical abuse, formal discipline	41.99			
Witnessed verbal or physical abuse	48.68			
Difficulty accessing medical care	41.58			
Difficulty accessing psychological/mental health services	20.89			
Index of conditions of confinement	2.66	1.54	0.00	6.00
*Covariates*				
Male	91.68			
Age (years)	37.08	9.20	18.59	68.93
High school degree or higher	43.41			
Hispanic/Latina/o ethnicity	85.19			
Speaks English well	52.74			
Undocumented	71.81			
Had health insurance pre-detention	40.97			
Detention facility 1 (privately operated)	43.00			
Detention facility 2 (county jail)	22.52			
Detention facility 3 (local jail)	13.18			
Detention facility 4 (county jail)	21.30			
Months detained by ICE (at time of survey)	8.70	4.68	3.29	55.53
Total incarceration prior to detention (months)	21.97	47.57	0.00	600.00

Source: authors’ analysis of data from the 2013–2014 Rodriguez Survey.

With regard to conditions of confinement, 74.9% of respondents reported experiencing sleep deprivation, 37.9% had difficulty seeing family, 42.0% experienced physical or verbal abuse or formal discipline, 48.7% witnessed other detained people experiencing physical or verbal abuse, 41.6% had difficulty accessing medical care, and 20.9% had difficulty accessing mental health services. On average, respondents experienced 2.7 of these conditions (SD 1.5, range 0–6). The majority reported good overall health (69.4%), worsening health in detention (56.0%), and no diagnosis of a mental health condition (83.6%). The average number of stress symptoms was 5.84 (SD 3.2, range 0–11).

Table 2 presents the coefficients of each model (Models 1–3 presenting odds ratios from logistic regression and Model 4 presenting incidence rate ratios from Poisson regression) testing the association between each individual condition and the sum of confinement conditions and the health outcomes, adjusting for covariates (see Appendix Tables 3, 4, 5, and 6 for full models). We found that each condition of confinement was independently associated with one or more negative health outcomes. For example, sleep deprivation, difficulty accessing medical services, and difficulty accessing mental health services were associated with poorer overall health, worse health in detention, and higher stress. There was also a cumulative effect to these conditions. For each additional confinement condition, the odds of reporting good health decreased by 24%, the odds of worsening general health rose by 39%, the odds of having had a mental health diagnosis increased by 25%, and the incidence rate ratio of stress symptoms rose by 0.15 for each additional condition in the index.

**Table 2:** Regression coefficients confinement conditions for various health outcomes (*N* = 493)

Confinement condition models	Good overall health (odds ratios)	Worse health in detention (odds ratios)	Diagnosed mental health condition (odds ratios)	Stress index (incidence rate ratios)
Experienced sleep deprivation	0.41***	2.87***	2.41*	1.77***
	(0.11)	(0.65)	(0.87)	(0.09)
Difficulty seeing family	1.00	1.92**	1.00	1.22***
	(0.21)	(0.39)	(0.27)	(0.05)
Experienced verbal or physical abuse, formal discipline	0.76	1.16	1.62^+^	1.17***
	(0.16)	(0.23)	(0.44)	(0.05)
Witnessed verbal or physical abuse	0.79	1.13	1.16	1.16***
	(0.17)	(0.22)	(0.31)	(0.05)
Difficulty accessing medical care	0.44***	2.19***	1.16	1.24***
	(0.09)	(0.44)	(0.30)	(0.05)
Difficulty accessing psychological/mental health services	0.41***	2.13**	2.68***	1.39***
	(0.10)	(0.54)	(0.79)	-0.06
Index of conditions of confinement	0.76***	1.39***	1.25*	1.15***
	(0.05)	(0.10)	(0.11)	(0.01)

Source: authors’ analysis of data from the 2013–2014 Rodriguez Survey. Note: standard errors are in parentheses. Each model controls for sex, age, education, ethnicity, English language ability, immigration status, pre-detention health insurance, detention facility, length of ICE detention, and length of total incarceration prior to detention. Models 1–3 use logistical regression and Model 4 uses Poisson regression. Full set of regression results with covariates in Tables 3, 4, 5 and 6 in the Appendix. ***p < 0.001, **p < 0.01, ^*^p < 0.05, and ^+^*p* < 0.1

**Fig. 1:**
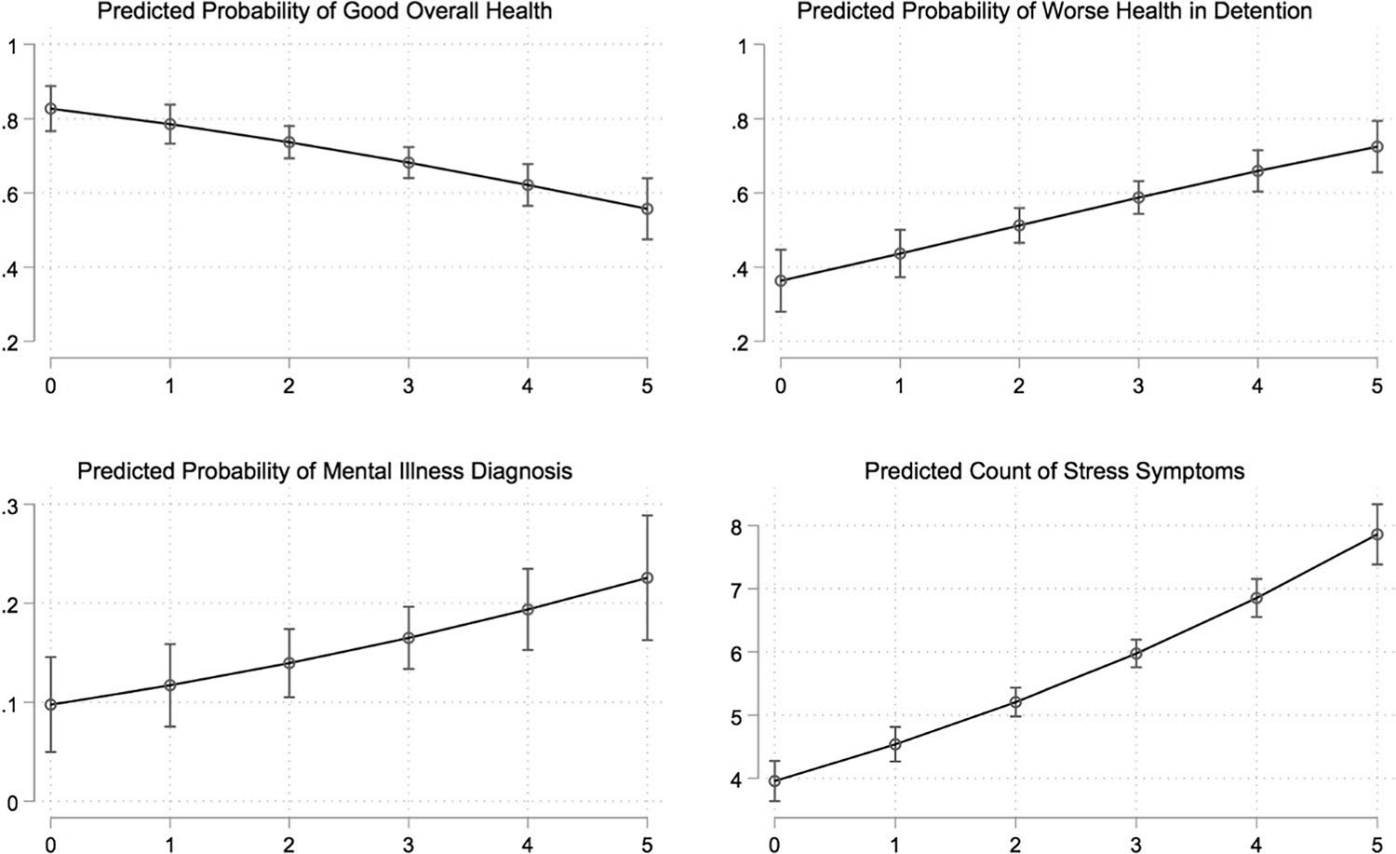
Predicted Health Outcomes by Index of Confinement Conditions

Figure 1 displays predicted probabilities of each outcome across each level of the index of conditions of confinement. For those who experienced a greater number of conditions of confinement, there were lower predicted probabilities of good health and higher predicted probabilities of worse health following detention. Similarly, those who experienced a greater number of conditions of confinement also had higher predicted probabilities of ever having been diagnosed with a mental illness and a higher predicted count of stress symptoms. For example, those who reported five compared to zero conditions of confinement had twice the predicted stress count (8.0 versus 3.9).

## Discussion

This study examined how conditions of confinement in immigration prison, individually and cumulatively, are associated with psychological stress, mental health, and general health. These conditions of confinement included sleep deprivation, social isolation from family via difficulty accessing family visitation, witnessing or experiencing abuse or harassment, and barriers to needed physical and mental health care. Respondents reported experiencing an average of three confinement conditions while detained. Our findings indicate that the experience of a single condition of confinement was associated with at least one poor health outcome. Overall, however, the cumulative impact of a greater number of conditions of confinement was associated with worsening general health, greater likelihood of mental health condition diagnosis, and increased stress symptoms. Statistical models that assess conditions in isolation may underestimate harms on health as single conditions may not yield statistically significant results on their own, but may “become significant” when combined with other conditions. Our findings underscore the importance of understanding immigrants’ experiences of conditions of confinement across multiple aspects of incarceration, including isolation, abuse, and poor medical and/or psychological care.

Our study builds on previous literature in multiple critical ways. Previous research on individuals in immigration prisons are predominantly based on studies conducted outside of the USA and focused on the extent and prevalence of mental health conditions among, primarily, asylum seekers with significant pre-migratory trauma. For example, in a systematic review of mental health conditions among detained immigrants involving 26 studies, only 4 were from the USA. These international studies previously documented high levels of anxiety, depression, PTSD, and other indicators of poor mental health [15]. Our study is also among the first to provide evidence that general and mental health outcomes are associated with experiences of the conditions of confinement in detention centers rather than solely attributable to pre-migratory trauma. While pre-migratory trauma and psychiatric comorbidity may increase an individual’s vulnerability to harms encountered in immigration prison, our findings indicate that the impact of conditions of confinement may be experienced by all detained individuals, not solely asylum seekers. Further, our findings offer insight into specific characteristics of the immigration prison context (e.g., denial of health care, exposure to verbal or physical abuse) that may contribute to adverse health outcomes. Previous studies have also found that deterioration of mental health was associated with increased duration in detention [16–18], level of perceived support from detention staff [19], and isolation from family [20]. Other existing ethnographic and theoretical literature has highlighted how adverse health outcomes in this context may represent a “syndemic,” or *synergistic epidemic*, where disease clusters may occur through biological interactions between diseases (e.g., mental health and physical health conditions) and social condition interactions (e.g., conditions of immigration detention) [21, 22]. By evaluating a wide range of conditions of confinement to understand the health impact of cumulative or co-occurring conditions, we offer an empirical view of how immigration prisons can harm health.

Our study also builds on research pertaining to the health impacts of prison conditions in the U.S.A by providing evidence that conditions of confinement in immigration prisons are associated with risk of worse health outcomes. Previous literature has focused on prisons in the criminal law context, finding associations between witnessed and experienced abuse, sleep conditions, and severing of social ties and poor health outcomes [23, 24]. However, few studies have empirically examined these conditions cumulatively or in U.S. immigration prisons. Our findings not only suggest that conditions of confinement cannot be addressed simply by removing or eliminating one single practice in immigration prisons—as these conditions occur cumulatively—but also affirm that immigration prisons replicate prison conditions and, therefore, are similarly associated with negative health outcomes because of their de facto design and operation. In other words, immigration detention facilities are punitive in their practice even if they are governed by civil rather than criminal law.

Despite the similarities, future research is still needed to explore the potentially unique health impact of incarceration under immigration, compared to criminal law. For example, individuals held in immigration prisons do not complete time-bound sentences and, in many cases, can be held indefinitely. Studies of detained immigrants in Canada and the United Kingdom (UK) have found that waiting for an indeterminate period for case adjudication, and perceived lack of control over this discretionary decision, can be detrimental to health [25, 26]. In the USA, there are very few constitutional limits on the length of immigration imprisonment; it is therefore possible that the uncertainty of prolonged and indefinite imprisonment may uniquely impact health of U.S.-based immigrants. Further research is needed to better understand the role of uncertainty in this particularly punitive context, which may be compounded by the absence of a right to representation unique to the immigration context and differs from the criminal context [27]. Notably, punitive detention conditions including those not captured in this study (e.g., holding cells often referred to as “hierleras” or “ice boxes” that are kept at very low temperatures) are often incorporated deliberately by anti-immigrant policymakers as a tool for demoralization and “attrition through enforcement” to encourage self-deportation [28, 29]. Understanding the long-term psychological harms of immigration detention should also incorporate the roles of demoralization and changes to self-concept and self-efficacy, which can independently be associated with adverse health outcomes including but not limited to psychiatric disorders [30, 31].

Future research could also incorporate pre-migratory factors alongside post-migratory factors, including those faced in immigration detention, to understand cumulative trauma across the migratory experience [5]. For example, conditions of confinement may be reminiscent of contexts in countries of origin, triggering re-traumatization or compounding mental health sequelae faced by individuals in immigration prisons. Similarly, U.S. immigration enforcement practices precede or proceed conditions of confinement in detention; i.e., workplace raids alone have been shown to be detrimental to health [32, 33]. Understanding trauma cumulatively across the continuum of migration experiences, as well as across varied immigration enforcement practices, is critical to capturing how multiple traumatic events exert negative influences on health, particularly with prior studies demonstrating a dose–response relationship whereby each additional traumatic event increases risk of multiple adverse health outcomes like mental illness and suicidal behaviors [34].

Lastly, further studies are needed to observe the enduring impact of conditions of confinement in the short- and long-term following release. In a mixed-methods study of adult refugees about 3 years following their release from immigration prisons in Australia, refugees continued to report an ongoing sense of insecurity, injustice, difficulty with relationships, profound changes to view of self, poor mental health, depression, memory disturbances, and persistent anxiety [30]. These are experiences likely to be felt by immigrants in the USA, where the social and political climate towards immigrants and people of color could influence reintegration of detained individuals into society.

### Limitations

Our study sample may not represent present-day detained populations. Immigration prison conditions worsened under the Trump administration’s changes to National Detention Standards, and the detention population increasingly includes more recent arrivals and asylum seekers and individuals without criminal convictions. However, this study may serve as a warning of harms to detained immigrants regardless of background characteristics. That is, this sample skews towards those with criminal records by virtue of all being members of the Rodriguez class action litigation and therefore subject to mandatory detention. Since this class action litigation, mandatory detention has been expanded to individuals without criminal convictions thereby portending harms to larger groups of immigrants. Relatedly, our largely Hispanic sample may not capture differences in experiences of conditions of confinement among other immigrant groups. For example, previous studies have found that detained immigrants from Africa and the Caribbean are over-represented in solitary confinement [35]. Moreover, this solely adult sample also does not capture unique harms that immigration imprisonment may have on children and youth. Second, our sampling design and cross-sectional analysis preclude elucidation of causal relationships. For the mental health diagnosis in particular, there may be a bidirectional relationship such that conditions of confinement increased the odds of having a mental health diagnosis and/or those with a diagnosis were more likely to experience a poor confinement condition. These limitations notwithstanding, our study not only helps to shed light on immigration prisons and their impacts on health and to identify the potential mechanisms of harm, but also helps in developing responses to mitigate these harms.

### Public Health Implications

Policies governing specific conditions—e.g., verbal harassment, continuous light [36], or solitary confinement—may address aspects of the detention experience, but our results show that individuals in immigration prison confront conditions of confinement cumulatively. Policymakers must urgently consider alternatives to detention and/or ending the practice of immigration detention that would remove conditions of confinement collectively. Evidence shows that immigrants fare better in community contexts than imprisonment; one study of UK asylum seekers in immigration prison versus the community found that detained asylum seekers experienced greater levels of depression, anxiety, and PTSD than the community comparison group [37]. There may also be household benefits given the impact of incarceration and immigration detention on entire family units [38], further bolstering the need to consider alternatives to imprisonment. Release from immigration detention in the context of the COVID-19 pandemic serves as an opportunity to document anticipated improved health outcomes for this population at an individual and household level.

Existing empirical evidence suggests that the legally recognized purpose of detention—to ensure compliance with immigration proceedings—may be overly punitive. Indeed, compliance with immigration court hearings is similar among immigrants in community contexts rather than imprisonment [39]. A study of immigration court data from 2008 to 2018 found that nearly 90% of all immigrants in immigration court with completed or pending removal cases attended all their court hearings; there was still high and majority compliance of greater than 80% when analysis was limited to previously detained cases [39]. In the absence of compliance as a reason for immigration imprisonment, the deleterious health impacts of immigration prisons are amplified and make alternative to detention programs even more urgent.

Notably, even within existing performance standards regarding the conditions of confinement, there is lack of oversight and accountability in the immigration detention system when detention facility contractors fail to meet performance standards. This has been extensively documented by the U.S. Department of Homeland Security Office of Inspector General [40]. As a result, specific piecemeal reforms to improve conditions are also unlikely to work as there is no mechanism for accountability. Harm reduction strategies in the immigration detention context must center oversight and accountability as central to mitigating the adverse consequences of these punitive, carceral-like, and often-substandard conditions [41]. Lastly, approximately three-quarters of immigrants are held in for-profit private facilities, where individuals are often held longer and in worse conditions of confinement as a result of cost-saving and profit-maximizing measures [42]. Therefore, ending contracts with private prison contractors represents another harm reduction strategy along a pathway that prioritizes decarceration and ending immigration detention [41, 42].

Conditions of confinement are associated with a range of poor mental and physical health outcomes among immigrants detained in immigration prisons in the USA, with multiple conditions of confinement further increasing the risk of poor health outcomes. Our findings provide evidence of the detrimental and cumulative impact of immigration prison on health and indicate that detention can harm health via multiple potential pathways. Policies that seek to improve specific conditions in detention centers may remove some risks of harm. Policy responses that consider alternatives to detention are likely to be most effective.

## Data Availability

The dataset analyzed during the current study is not publicly available due sensitivity of the data and populations. Researchers interested in access to the data may contact Dr. Caitlin Patler (patler@ucdavis.edu) who is able to field requests for use of this data on an individual basis.

## Appendix

Methods

Study Sample: To assess the relative comparability of the Rodriguez Survey (RS) to individuals detained during a similar time period, we examined data from ICE, acquired by the Transactional Records Access Clearinghouse at Syracuse University, which contains limited demographic information on all people detained by ICE during FY 2015 (October 2014 through September 2015), the only year for which such data exist for public use [5]. We restricted the TRAC data to adult immigrants detained in California for 180 days or more (*n* = 481) and found the following: 93.7% of the TRAC sample was identified as male, compared to 91.7% of the RS sample; the mean age in the TRAC data was 33.1 years old, compared to 37.1 years in the RS; 62.9% of the TRAC samples were from Mexico, Central America, or South America, compared to 89.9% in the RS; and the average length of ICE detention was 8.7 months in the TRAC data, compared to 8.7 months in the RS.

**Table 3:** Odds ratios from logistic regression analysis of good overall health, by various confinement conditions and covariates

	Good overall health	Good overall health	Good overall health	Good overall health	Good overall health	Good overall health	Good overall health
Experienced sleep deprivation	0.41***						
	(0.11)						
Difficulty seeing family		1.00					
		(0.21)					
Experienced verbal or physical abuse, formal discipline			0.76				
			(0.16)				
Witnessed verbal or physical abuse				0.79			
				(0.17)			
Difficulty accessing medical care					0.44***		
					(0.09)		
Difficulty accessing psychological/mental health services						0.41***	
						(0.10)	
Index of conditions of confinement							0.76***
							(0.05)
Male	1.66	1.82	1.73	1.75	1.62	1.42	1.33
	(0.70)	(0.77)	(0.73)	(0.74)	(0.69)	(0.61)	(0.57)
Age (years)	0.97*	0.97*	0.97**	0.97*	0.97*	0.97*	0.97**
	(0.01)	(0.01)	(0.01)	(0.01)	(0.01)	(0.01)	(0.01)
High school degree or higher	0.99	0.99	1.02	1.01	0.97	1.14	1.05
	(0.22)	(0.22)	(0.22)	(0.22)	(0.22)	(0.26)	(0.24)
Hispanic/Latina/o ethnicity	0.61	0.64	0.63	0.64	0.62	0.65	0.61
	(0.19)	(0.20)	(0.19)	(0.20)	(0.19)	(0.20)	(0.19)
Speaks English well	0.68	0.75	0.76	0.77	0.78	0.75	0.78
	(0.16)	(0.18)	(0.18)	(0.18)	(0.19)	(0.18)	(0.19)
Undocumented	1.04	1.00	0.98	0.99	0.98	1.05	1.02
	(0.26)	(0.25)	(0.24)	(0.25)	(0.25)	(0.26)	(0.26)
Had health insurance pre-detention	1.04	0.94	0.94	0.95	0.93	0.92	0.99
	(0.22)	(0.20)	(0.20)	(0.20)	(0.20)	(0.20)	(0.21)
Detention facility 2 (county jail; ref: private facility)	1.72^+^	1.68^+^	1.66^+^	1.64^+^	1.50	1.55	1.51
	(0.50)	(0.49)	(0.48)	(0.48)	(0.44)	(0.46)	(0.45)
Detention facility 3 (local jail; ref: private facility)	1.45	1.53	1.53	1.44	1.38	1.52	1.33
	(0.55)	(0.57)	(0.57)	(0.54)	(0.52)	(0.58)	(0.50)
Detention facility 4 (county jail; ref: private facility)	1.25	1.18	1.20	1.23	1.08	1.12	1.21
	(0.34)	(0.31)	(0.32)	(0.33)	(0.29)	(0.30)	(0.33)
Months detained by ICE (at time of survey)	0.97	0.98	0.98	0.98	0.98	0.98	0.98
	(0.02)	(0.02)	(0.02)	(0.02)	(0.02)	(0.02)	(0.02)
Logged total incarceration prior to detention (months)	0.95	0.95	0.95	0.95	0.94	0.96	0.96
	(0.08)	(0.08)	(0.08)	(0.08)	(0.08)	(0.08)	(0.08)
Constant	16.63***	7.67*	9.64**	8.87**	13.42**	10.71**	23.61***
	(14.12)	(6.26)	(7.93)	(7.24)	(11.18)	(8.83)	(20.54)
Observations	493	493	493	493	493	493	493

Note: standard errors are in parentheses. ****p* < 0.001, ***p* < 0.01, **p* < 0.05, and ^+^*p* < 0.1

**Table 4:** Odds ratios from logistic regression analysis of worse health in detention, by various confinement conditions and covariates

	Worse health in detention	Worse health in detention	Worse health in detention	Worse health in detention	Worse health in detention	Worse health in detention	Worse health in detention
Experienced sleep deprivation	2.87***						
	(0.65)						
Difficulty seeing family		1.92**					
		(0.39)					
Experienced verbal or physical abuse, formal discipline			1.16				
			(0.23)				
Witnessed verbal or physical abuse				1.13			
				(0.22)			
Difficulty accessing medical care					2.19***		
					(0.44)		
Difficulty accessing psychological/mental health services						2.13**	
						(0.54)	
Index of conditions of confinement							1.39***
							(0.10)
Male	1.49	1.49	1.32	1.31	1.47	1.60	1.98
	(0.61)	(0.61)	(0.53)	(0.52)	(0.60)	(0.65)	(0.83)
Age (years)	1.05***	1.05***	1.05***	1.05***	1.05***	1.05***	1.05***
	(0.01)	(0.01)	(0.01)	(0.01)	(0.01)	(0.01)	(0.01)
High school degree or higher	0.98	1.04	0.97	0.97	0.99	0.89	0.92
	(0.21)	(0.22)	(0.20)	(0.20)	(0.21)	(0.19)	(0.20)
Hispanic/Latina/o ethnicity	0.66	0.66	0.65	0.64	0.66	0.62^+^	0.66
	(0.19)	(0.19)	(0.19)	(0.18)	(0.19)	(0.18)	(0.20)
Speaks English well	0.71	0.60*	0.63*	0.63*	0.60*	0.63*	0.60*
	(0.16)	(0.14)	(0.14)	(0.14)	(0.14)	(0.14)	(0.14)
Undocumented	1.48	1.50^+^	1.54^+^	1.53^+^	1.56^+^	1.49^+^	1.55^+^
	(0.36)	(0.36)	(0.37)	(0.37)	(0.38)	(0.36)	(0.38)
Had health insurance pre-detention	1.19	1.31	1.34	1.32	1.35	1.35	1.27
	(0.25)	(0.27)	(0.27)	(0.27)	(0.28)	(0.27)	(0.26)
Detention facility 2 (county jail; ref: private facility)	1.05	1.09	1.07	1.07	1.20	1.14	1.25
	(0.28)	(0.29)	(0.28)	(0.28)	(0.32)	(0.30)	(0.33)
Detention facility 3 (local jail; ref: private facility)	1.15	1.09	1.06	1.09	1.20	1.07	1.28
	(0.41)	(0.38)	(0.37)	(0.38)	(0.43)	(0.37)	(0.46)
Detention facility 4 (county jail; ref: private facility)	0.88	0.97	0.93	0.92	1.04	0.98	0.91
	(0.23)	(0.25)	(0.23)	(0.23)	(0.27)	(0.25)	(0.23)
Months detained by ICE (at time of survey)	0.98	0.98	0.98	0.98	0.98	0.98	0.98
	(0.02)	(0.02)	(0.02)	(0.02)	(0.02)	(0.02)	(0.02)
Logged total incarceration prior to detention (months)	1.02	1.00	1.02	1.02	1.03	1.01	1.01
	(0.08)	(0.08)	(0.08)	(0.08)	(0.08)	(0.08)	(0.08)
Constant	0.10**	0.15*	0.22^+^	0.23^+^	0.15*	0.19*	0.07**
	(0.08)	(0.12)	(0.17)	(0.18)	(0.12)	(0.15)	(0.06)
Observations	493	493	493	493	493	493	493

Note: standard errors are in parentheses. ****p* < 0.001, ***p* < 0.01, **p* < 0.05, and ^+^*p* < 0.1

**Table 5:** Odds ratios from logistic regression analyses of mental health condition diagnosis, by various confinement conditions and covariates

	Diagnosed with depression or other mental health condition	Diagnosed with depression or other mental health condition	Diagnosed with depression or other mental health condition	Diagnosed with depression or other mental health condition	Diagnosed with depression or other mental health condition	Diagnosed with depression or other mental health condition	Diagnosed with depression or other mental health condition
Experienced sleep deprivation	2.41*						
	(0.87)						
Difficulty seeing family		1.00					
		(0.27)					
Experienced verbal or physical abuse, formal discipline			1.62^+^				
			(0.44)				
Witnessed verbal or physical abuse				1.16			
				(0.31)			
Difficulty accessing medical care					1.16		
					(0.30)		
Difficulty accessing psychological/mental health services						2.68***	
						(0.79)	
Index of conditions of confinement							1.25*
							(0.11)
Male	0.10***	0.10***	0.10***	0.10***	0.10***	0.13***	0.12***
	(0.06)	(0.06)	(0.06)	(0.06)	(0.06)	(0.08)	(0.07)
Age (years)	1.01	1.01	1.02	1.01	1.01	1.01	1.02
	(0.01)	(0.01)	(0.01)	(0.01)	(0.01)	(0.01)	(0.01)
High school degree or higher	1.36	1.33	1.31	1.33	1.34	1.11	1.29
	(0.39)	(0.38)	(0.37)	(0.37)	(0.38)	(0.33)	(0.37)
Hispanic/Latina/o ethnicity	1.06	1.02	1.04	1.02	1.02	0.99	1.03
	(0.38)	(0.36)	(0.37)	(0.36)	(0.36)	(0.36)	(0.37)
Speaks English well	1.63	1.50	1.45	1.48	1.48	1.44	1.42
	(0.53)	(0.48)	(0.46)	(0.47)	(0.47)	(0.47)	(0.45)
Undocumented	0.89	0.91	0.97	0.92	0.91	0.83	0.91
	(0.27)	(0.28)	(0.30)	(0.28)	(0.28)	(0.26)	(0.28)
Had health insurance pre-detention	1.90*	2.08**	2.15**	2.07**	2.09**	2.13**	2.04**
	(0.52)	(0.57)	(0.59)	(0.56)	(0.57)	(0.59)	(0.56)
Detention facility 2 (county jail; ref: private facility)	0.26**	0.27**	0.26**	0.27**	0.27**	0.30*	0.28**
	(0.13)	(0.13)	(0.13)	(0.13)	(0.13)	(0.14)	(0.14)
Detention facility 3 (local jail; ref: private facility)	0.17**	0.16**	0.16**	0.17**	0.17**	0.18**	0.19**
	(0.11)	(0.10)	(0.10)	(0.11)	(0.10)	(0.11)	(0.12)
Detention facility 4 (county jail; ref: private facility)	0.74	0.79	0.76	0.77	0.80	0.84	0.77
	(0.25)	(0.26)	(0.25)	(0.26)	(0.26)	(0.28)	(0.26)
Months detained by ICE (at time of survey)	1.05^+^	1.05^+^	1.05^+^	1.05^+^	1.05^+^	1.05^+^	1.05^+^
	(0.03)	(0.03)	(0.03)	(0.03)	(0.03)	(0.03)	(0.03)
Logged total incarceration prior to detention (months)	1.21^+^	1.21^+^	1.21^+^	1.21^+^	1.21^+^	1.19^+^	1.20^+^
	(0.13)	(0.13)	(0.13)	(0.13)	(0.13)	(0.13)	(0.12)
Constant	0.15^+^	0.32	0.21	0.29	0.29	0.22	0.13^+^
	(0.16)	(0.33)	(0.21)	(0.30)	(0.30)	(0.23)	(0.14)
Observations	493	493	493	493	493	493	493

Note: standard errors are in parentheses. ****p* < 0.001, ***p* < 0.01, **p* < 0.05, and ^+^*p* < 0.1

**Table 6:** Incidence rate ratios from Poisson regression analyses of stress index, by various confinement conditions and covariates

	Count of stress symptoms	Count of stress symptoms	Count of stress symptoms	Count of stress symptoms	Count of stress symptoms	Count of stress symptoms	Count of stress symptoms
Experienced sleep deprivation	1.77***						
	(0.09)						
Difficulty seeing family		1.22***					
		(0.05)					
Experienced verbal or physical abuse, formal discipline			1.17***				
			(0.05)				
Witnessed verbal or physical abuse				1.16***			
				(0.05)			
Difficulty accessing medical care					1.24***		
					(0.05)		
Difficulty accessing psychological/mental health services						1.39***	
						(0.06)	
Index of conditions of confinement							1.15***
							(0.01)
Male	0.84*	0.83*	0.82**	0.82**	0.82**	0.88^+^	0.93
	(0.06)	(0.06)	(0.06)	(0.06)	(0.06)	(0.07)	(0.07)
Age (years)	1.01*	1.01***	1.01***	1.01**	1.01**	1.01**	1.01***
	(0.00)	(0.00)	(0.00)	(0.00)	(0.00)	(0.00)	(0.00)
High school degree or higher	1.05	1.07^+^	1.04	1.04	1.06	1.00	1.02
	(0.04)	(0.04)	(0.04)	(0.04)	(0.04)	(0.04)	(0.04)
Hispanic/Latina/o ethnicity	1.01	1.00	0.99	0.98	0.99	0.98	1.00
	(0.06)	(0.06)	(0.06)	(0.05)	(0.05)	(0.05)	(0.06)
Speaks English well	1.08^+^	1.00	1.01	1.00	1.00	1.01	1.00
	(0.05)	(0.04)	(0.04)	(0.04)	(0.04)	(0.05)	(0.04)
Undocumented	1.05	1.06	1.09^+^	1.08	1.08	1.06	1.07
	(0.05)	(0.05)	(0.05)	(0.05)	(0.05)	(0.05)	(0.05)
Had health insurance pre-detention	1.00	1.05	1.07^+^	1.06	1.07	1.07^+^	1.04
	(0.04)	(0.04)	(0.04)	(0.04)	(0.04)	(0.04)	(0.04)
Detention facility 2 (county jail; ref: private facility)	0.87*	0.89*	0.89*	0.89*	0.91^+^	0.91^+^	0.93
	(0.05)	(0.05)	(0.05)	(0.05)	(0.05)	(0.05)	(0.05)
Detention facility 3 (local jail; ref: private facility)	1.14^+^	1.11	1.10	1.14*	1.13^+^	1.11	1.18*
	(0.08)	(0.07)	(0.07)	(0.08)	(0.08)	(0.07)	(0.08)
Detention facility 4 (county jail; ref: private facility)	1.00	1.05	1.02	1.01	1.06	1.06	1.02
	(0.05)	(0.05)	(0.05)	(0.05)	(0.05)	(0.05)	(0.05)
Months detained by ICE (at time of survey)	1.00	1.00	1.00	1.00	1.00	1.00	1.00
	(0.00)	(0.00)	(0.00)	(0.00)	(0.00)	(0.00)	(0.00)
Logged total incarceration prior to detention (months)	1.02	1.02	1.02	1.02	1.02	1.02	1.02
	(0.02)	(0.02)	(0.02)	(0.02)	(0.02)	(0.02)	(0.02)
Constant	3.03***	4.24***	4.27***	4.45***	4.28***	4.32***	2.86***
	(0.46)	(0.63)	(0.64)	(0.66)	(0.63)	(0.64)	(0.44)
Observations	493	493	493	493	493	493	493

Note: standard errors are in parentheses. ****p* < 0.001, ***p* < 0.01, **p* < 0.05, ^+^*p* < 0.1

## References

[1] Patler C, Golash-Boza TM (2017). The fiscal and human costs of immigrant detention and deportation in the United States. Sociol Compass.

[2] Transactional Records Access Clearinghouse., “Tracking over 2 million ICE arrests: a first look,” 2018. https://trac.syr.edu/immigration/reports/529/. Accessed 21 Nov 2018.

[3] Lopez MH, Gonzalez-Barrera A, and Krogstad JM, “Hispanics and their views of immigration reform,” *Pew Research Center’s Hispanic Trends Project*, Oct. 29, 2014. https://www.pewresearch.org/hispanic/2014/10/29/chapter-5-hispanics-and-their-views-of-immigration-reform/. Accessed 04 Sept 2019.

[4] Perreira KM, Pedroza JM (2019). Policies of exclusion: implications for the health of immigrants and their children. Annu Rev Public Health.

[5] Saadi A, Young MEDT, Patler C, Estrada JL, and Venters HD, “Understanding US immigration detention: reaffirming rights and addressing social-structural determinants of health,” *Health and Human Rights Journal*, May 2020, [Online]. Available: https://www.hhrjournal.org/2020/05/understanding-us-immigration-detention-reaffirming-rights-and-addressing-social-structural-determinants-of-health/. Accessed 27 Jul 2020. PMC7348446 32669800

[6] Massoglia M, Pridemore WA (2015). Incarceration and health. Annu Rev Sociol.

[7] Brinkley-Rubinstein L (2013). Incarceration as a catalyst for worsening health. Health & Justice.

[8] Longazel J, Berman J, Fleury-Steiner B (2016). The pains of immigrant imprisonment: pains of immigrant imprisonment. Sociol Compass.

[9] Human Rights Watch (Organization) (2018). American Civil Liberties Union, National Immigrant Justice Center, and Detention Watch Network, Eds., Code red: the fatal consequences of dangerously substandard medical care in immigration detention.

[10] American Civil Liberties Union, Detention Watch Network, and National Immigrant Justice Center, “Fatal neglect: how ICE ignores death in detention,” Feb. 2016. [Online]. Available: https://www.aclu.org/report/fatal-neglect-how-ice-ignores-death-detention. Accessed 19 Apr 2019.

[11] Patler C and Saadi A, “Risk of poor outcomes with COVID-19 among U.S. detained immigrants: a cross-sectional study,” *J Immigr Minor Health*, Mar. 2021, 10.1007/s10903-021-01173-z. 10.1007/s10903-021-01173-z PMC7930890 33661415

[12] Patler C and Branic N, “Patterns of family visitation during immigration detention,” *RSF: The Russell Sage Foundation Journal of the Social Sciences*, vol. 3, no. 4, pp. 18–36, Jul. 2017, 10.7758/RSF.2017.3.4.02.

[13] Western B, Braga A, Kohl R (2017). A longitudinal survey of newly-released prisoners: methods and design of the Boston Reentry Study 1. Fed Probat.

[14] American Psychological Association, “Stress in America: missing the health care connection,” Washington, D.C., 2013. [Online]. Available: https://www.apa.org/news/press/releases/stress/2012/full-report.pdf. Accessed 07 Jun 2021.

[15] von Werthern M *et al.*, “The impact of immigration detention on mental health: a systematic review,” *BMC Psychiatry*, vol. 18, Dec. 2018, 10.1186/s12888-018-1945-y. 10.1186/s12888-018-1945-y PMC6282296 30522460

[16] Keller AS (2003). Mental health of detained asylum seekers. Lancet.

[17] Young P, Gordon MS (2016). Mental health screening in immigration detention: a fresh look at Australian government data. Australas Psychiatry.

[18] Green JP and Eagar K, “The health of people in Australian immigration detention centres,” *Med. J. Aust.*, vol. 192, no. 2, Jan. 2010, [Online]. Available: https://www.mja.com.au/journal/2010/192/2/health-people-australian-immigration-detention-centres 10.5694/j.1326-5377.2010.tb03419.x 20078404

[19] Puthoopparambil S. J., Bjerneld M., and Källestål C., “Quality of life among immigrants in Swedish immigration detention centres: a cross-sectional questionnaire study,” *Glob Health Action*, vol. 8, p. Jul. 2015, 10.3402/gha.v8.28321. 10.3402/gha.v8.28321 PMC4507748 26193898

[20] Steel Z, Silove D, Brooks R, Momartin S, Alzuhairi B, Susljik I (2006). Impact of immigration detention and temporary protection on the mental health of refugees. Br J Psychiatry.

[21] Carney M, Lerman S, Ostrach B, Singer M (2017). Sickness in the detention system: syndemics of mental distress, malnutrition, and immigration stigma in the United States. Stigma syndemics: new directions in biosocial health.

[22] Houston AR (2021). United States immigration detention amplifies disease interaction risk: a model for a transnational ICE-TB-DM2 syndemic. Glob Public Health.

[23] Lorek A (2009). The mental and physical health difficulties of children held within a British immigration detention center: a pilot study. Child Abuse Negl.

[24] Steel Z, Momartin S, Silove D, Coello M, Aroche J, Tay KW (2011). Two year psychosocial and mental health outcomes for refugees subjected to restrictive or supportive immigration policies. Soc Sci Med.

[25] Hasselberg I (2017). Enduring uncertainty: deportation, punishment and everyday life.

[26] Cleveland J, Kronick R, Gros H, Rousseau C (2018). Symbolic violence and disempowerment as factors in the adverse impact of immigration detention on adult asylum seekers’ mental health. Int J Public Health.

[27] Ryo E (2019). Understanding immigration detention: causes, conditions, and consequences. Annual Review of Law and Social Science.

[28] Park K-S, “Self-deportation nation,” *Harvard Law Review*, May 2019, 132, 1878-1941.

[29] Kobach K (2008). Attrition through enforcement: a rational approach to illegal immigration. Tulsa Journal of Comparative and International Law.

[30] Coffey GJ, Kaplan I, Sampson RC, Tucci MM (2010). The meaning and mental health consequences of long-term immigration detention for people seeking asylum. Soc Sci Med.

[31] Tecuta L, Tomba E, Grandi S, Fava GA (2015). Demoralization: a systematic review on its clinical characterization. Psychol Med.

[32] Lopez WD (2017). Health implications of an immigration raid: findings from a Latino community in the Midwestern United States. J Immigrant Minority Health.

[33] Hacker K (2011). The impact of Immigration and Customs Enforcement on immigrant health: perceptions of immigrants in Everett, Massachusetts, USA. Soc Sci Med.

[34] Fink DS, Galea S (2015). Life course epidemiology of trauma and related psychopathology in civilian populations. Curr Psychiatry Rep.

[35] Franco K, Patler C, and Reiter K, “Punishing status and the punishment status quo: solitary confinement in U.S. immigration prisons, 2013–2017,” *Punishment & Society*, p. 1462474520967804, Oct. 2020, 10.1177/1462474520967804.

[36] Wilson M., “Ninth circuit revives Ad Seg 24-hour lighting claim,” *Prison Legal News*, Oct. 10, 2014. https://www.prisonlegalnews.org/news/2014/oct/10/ninth-circuit-revives-ad-seg-24-hour-lighting-claim/. Accessed 07 June 2021.

[37] Robjant K, Hassan R, Katona C (2009). Mental health implications of detaining asylum seekers: systematic review. Br J Psychiatry.

[38] Patler C. and Gonzalez G., “Compounded vulnerability: the consequences of immigration detention for institutional attachment and system avoidance in mixed-immigration-status families,” *Social Problems*, no. spaa069, Dec. 2020, 10.1093/socpro/spaa069.

[39] Eagly I. V. and Shafer S., “Measuring in absentia removal in immigration court,” Social Science Research Network, Rochester, NY, SSRN Scholarly Paper ID 3633267, Mar. 2020. [Online]. Available: https://papers.ssrn.com/abstract=3633267. Accessed 07 Jun 2021.

[40] Office of Inspector General, Department of Homeland Security, “ICE does not fully use contracting tools to hold detention facility contractors accountable for failing to meet performance standards.” Jan. 29, 2019. [Online]. Available: https://www.oig.dhs.gov/sites/default/files/assets/2019-02/OIG-19-18-Jan19.pdf

[41] Physicians for Human Rights Asylum Policy Working Group, “Re-imagining the asylum system: recommendations from asylum medicine experts | Health Affairs Blog.” https://www.healthaffairs.org/do/10.1377/hblog20210510.133971/full/

[42] Saadi A, Tesema L (2019). Privatisation of immigration detention facilities. Lancet.

